# Skin pathology in ALS: Diagnostic implications and biomarker potential

**DOI:** 10.17305/bb.2025.12100

**Published:** 2025-04-02

**Authors:** Ying Gao, Yanchao Lu, Ranran Chen, Shumin Zhao, Jialing Liu, Sutian Zhang, Xue Bai, Jingjing Zhang

**Affiliations:** 1 Department of Neurology, Medical Research Center, Chifeng Municipal Hospital, Chifeng, China; 2 Chifeng Clinical Medical College of Inner Mongolia Medical University, Chifeng, China

**Keywords:** Amyotrophic lateral sclerosis, ALS, skin, biomarkers, diagnosis, protein aggregation

## Abstract

Amyotrophic lateral sclerosis (ALS) is a fatal neurodegenerative disease characterized by the loss of motor neurons in the spinal cord and brain, resulting in motor deficits and muscle atrophy. Approximately 5–10% of ALS patients are familial (fALS), while the rest are sporadic (sALS). Currently, early diagnosis of ALS cannot be achieved based on clinical manifestations and electromyography due to the lack of effective and easily available biomarkers. The skin and central nervous system (CNS) share the same embryonic origin. Several skin biomarkers have been found in many neurodegenerative diseases, such as abnormal deposition of pathological α-synuclein (α-Syn) in Parkinson’s disease. Thus, molecular changes in the skin associated with ALS-specific pathological events could readily be detected and become biomarkers for ALS through skin testing. Here, we summarize the literature on pathological changes in the skin of ALS patients and animal models, including structural abnormalities of the skin, reduced density of skin nerve fibers, abnormal protein aggregation, altered mitochondrial morphology and function, and dysregulation of skin inflammation, which may be useful for early diagnosis and monitoring of ALS progression.

## Introduction

**Figure 1.**
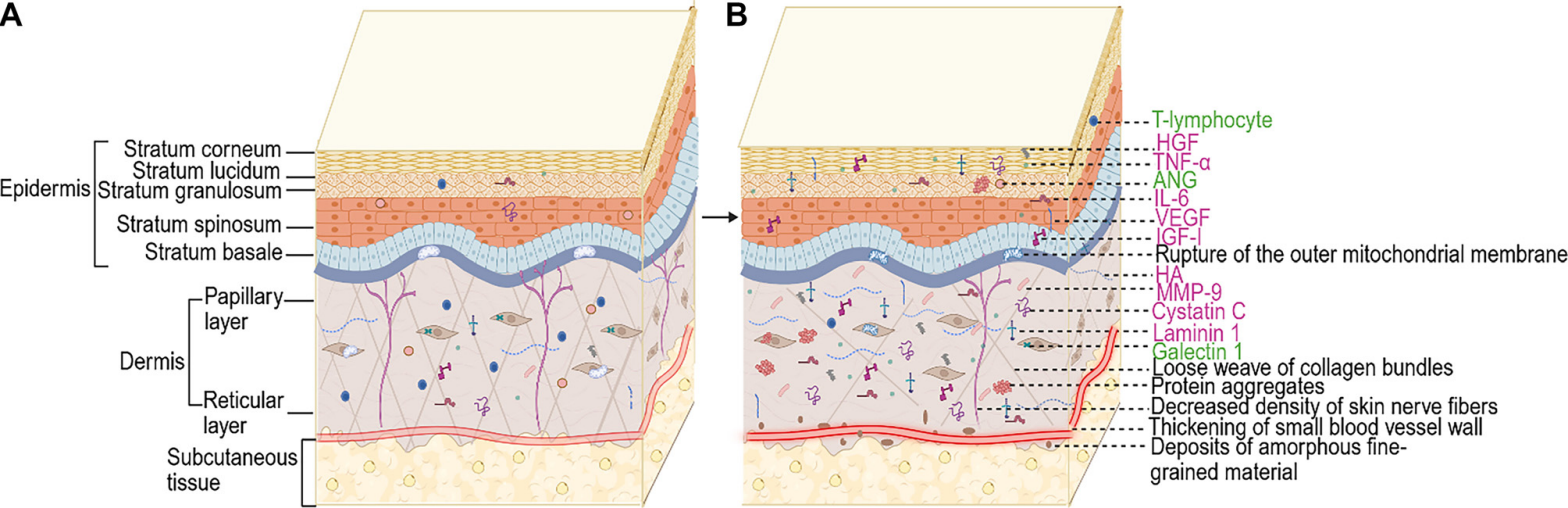
(A) Normal skin and (B) possible pathology in ALS skin. The pink text indicates increased molecules and the green text represents decreased molecules. The following changes occur in the skin of ALS patients compared to normal human skin: (1) Structural abnormalities include a reduced number of collagen bundles, sparse weaving and widening of gaps; deposition of amorphous fine-grained material; reduced density of nerve fibers; thickening of blood vessel walls and decreased expression of ANG. (2) Abnormal deposition of genetically determined ALS-related proteins, including superoxide dismutase 1 (SOD1), TAR DNA binding protein 43 (TDP-43), fused in sarcoma (FUS), valine-containing proteins (VCP), ubiquitin 2 (UBQLN2), vesicle-associated membrane protein-associated protein-B (VAPB), and progranulin (PGRN). (3) Abnormal mitochondrial morphology with reduced long-axis length, area, and circumference, rupture of the outer membrane, and cristae lysis. (4) Dysregulation of inflammatory response in the skin, with a decrease in the number of T lymphocytes (Tregs) and an increase in the expression of IL-6 and TNF-α. (5) Other molecules such as MMP-9, IGF-I, VEGF, HGF, cystatin C, laminin 1, and HA were increased, and galectin-1 was decreased in the skin of ALS patients. (Created by BioRender.com). Abbreviations: ALS: Amyotrophic lateral sclerosis; HGF: Hepatocyte growth factor; VEGF: Vascular endothelial growth factor; HA: Hyaluronic acid; ANG: Angiogenin; MMP-9: Matrix metalloproteinase 9; TNF-α: Tumor necrosis factor-α; IL-6: Interleukin-6; IGF-I: Insulin-like growth factor I.

Amyotrophic lateral sclerosis (ALS) is a degenerative disease with a poor prognosis, resulting from the loss of motor neurons (MNs) in the cerebral cortex and spinal cord. Most patients die of respiratory failure within 3–5 years of disease onset [1]. ALS includes both sporadic and familial forms, with familial ALS (fALS) accounting for approximately 10% of cases and sporadic ALS (sALS) comprising the remaining 90% [2]. To date, the U.S. Food and Drug Administration (FDA) has approved several drugs for ALS treatment, including riluzole, AMX0035, and tofersen. These drugs can slow disease progression but do not provide a cure [3]. Early diagnosis is essential for effective treatment of ALS. However, diagnostic challenges persist: spinal cord and brain tissues are not easily accessible, blood markers lack specificity, and cerebrospinal fluid (CSF) sampling carries increased risk of complications in patients with advanced disease [4]. A definitive diagnosis typically involves identifying the progressive spread of symptoms affecting the medulla oblongata, cervical, thoracic, and lumbar regions in both upper and lower MNs, using clinical and neurophysiological testing [5, 6]. Due to the lack of simple and effective early-stage diagnostic methods, formal diagnosis is often delayed by 10–16 months [7]. This highlights the urgent need for reliable biomarkers to facilitate early diagnosis and monitor disease progression. During weeks 3–4 of human embryonic development, both the skin and nervous system originate from the neuroectoderm. The epidermis forms from the ventral ectoderm, while the neural ectoderm develops along the dorsal side, eventually thickening into the neural plate and shaping into the neural tube. Because of this common origin, several key factors and regulatory mechanisms involved in the central and peripheral nervous systems are also active in the skin [8]. Epidermal keratinocytes play a central role in the connection between the skin and neural development. These cells share hormones and receptors with the central nervous system (CNS), including the N-methyl-D-aspartate (NMDA) receptor, which influences both epidermal proliferation and barrier maintenance, and is also involved in learning and memory regulation [9]. Furthermore, epidermal keratinocytes express components of the hypothalamic-pituitary-adrenal (HPA) axis that regulate the skin’s antimicrobial defense mechanisms [10]. These findings suggest a close relationship between the epidermal barrier and neurodevelopment. Given their shared embryonic origin, pathological changes in the nervous system may be detectable via skin biopsy [9]. Skin changes have been observed in several neurodegenerative diseases prior to the onset of neurological symptoms. For instance, α-synuclein (α-Syn) deposits in the skin may serve as a reliable biomarker for diagnosing Parkinson’s disease before neurological symptoms appear [11]. Patients with Alzheimer’s disease (AD) have also been found to exhibit a less acidic skin pH, increased skin hydration, and reduced skin elasticity compared to healthy controls [12]. The connection between morphological and biochemical changes in the skin and ALS is an emerging area of research with significant potential for advancing diagnostics. In this paper, we systematically review structural changes in the skin of ALS patients, related cellular alterations, molecular and biochemical skin changes, and associated models, with the aim of identifying potential skin biomarkers for the diagnosis and assessment of ALS (Figure 1).

## Changes in skin structure and appendages

Skin is the largest organ in the human body, covering the entire body surface and made up of the epidermis, dermis, subcutaneous tissue, and skin appendages. The epidermis is the outermost layer and is divided into five layers from the surface inward: the stratum corneum, stratum lucidum, stratum granulosum, stratum spinosum, and stratum basale. It primarily consists of keratinocytes, melanocytes, Langerhans cells, and Merkel cells [13]. Beneath the epidermis lies the dermis, which is mainly composed of extracellular matrix and fibroblasts. The dermis is further divided into the papillary and reticular layers. The papillary layer connects to the basal layer of the epidermis, while the reticular layer attaches to the subcutaneous tissue. This deeper tissue is rich in collagen fibers, elastin fibers, reticular fibers, and hyaluronic acid (HA). The dermis also contains numerous nerve endings, blood vessels, and lymphatic vessels [14]. The subcutaneous tissue has a loose structure and contains a large number of fat cells. The skin also secretes substances like sweat and sebum through the sweat and sebaceous glands, which are part of the skin’s appendages [13].

### Abnormalities in skin structure

Structural abnormalities in the dermis of ALS patients have been repeatedly observed. The skin of an ALS patient is often described as soft to the touch, resembling tanned leather, and exhibits the “delayed return phenomenon (DRP)” [15]. Collagen, a key structural protein in the skin’s connective tissue, provides mechanical support and protection. In ALS patients, increased collagen solubility and density, as well as altered cross-linking, have been reported. Specifically, there is a decrease in type IV collagen—primarily located in the basement membrane—and type I collagen, alongside an increase in type III procollagen in the dermis [16–18]. Light microscopy has revealed a reduced number of collagen bundles, which appear more loosely arranged, with progressively wider gaps in the connective tissue of ALS patients exhibiting DRP. Electron microscopy shows extensive deposition of amorphous, fine-grained material in the dermal matrix, with this deposition increasing as the disease progresses. This material can also accumulate on the surface of collagen fibers, leading to their separation and fragmentation, and contributing to further connective tissue abnormalities [19, 20]. Interestingly, a patient-derived ALS tissue-engineered skin model (ALS-TES) has demonstrated several structural abnormalities even in pre-symptomatic C9orf72-linked patients. These include epidermal undifferentiation, abnormalities at the dermal-epidermal junction, delamination, keratinocyte infiltration, and collagen disorganization [21].

### Decreased density of skin nerve fibers

In recent years, an increasing number of studies have shown that patients with ALS also experience a range of non-motor manifestations, including autonomic dysfunction and involvement of the sensory nervous system [22]. Small fiber neuropathy in the skin—reflected by both sensory and autonomic dysfunction—has been reported alongside motor damage in ALS patients [23]. Research has demonstrated that autonomic dysfunction in early-stage ALS may present as subclinical impairments in cardiovascular, sudomotor, gastrointestinal, salivary, and lacrimal regulation [24]. Sympathetic skin response (SSR) testing in ALS patients has shown prolonged latency and reduced amplitude, suggesting impaired sympathetic efferent function. Additionally, ultrasonography of the vagus nerve (VN) at the level of the thyroid gland has revealed a significant decrease in the VN’s cross-sectional area, which may indicate VN atrophy—further supporting the presence of dysautonomia in ALS patients [25]. Moreover, ALS patients have shown significantly higher autonomic-related COMPASS-31 scores and longer SSR latencies in the lower extremities than in the upper extremities, when compared with healthy controls [26, 27]. These findings support involvement of skin autonomic nerve fibers in ALS, which can manifest as abnormalities in cutaneous vasoconstriction, thermoregulation, and sweating. Histopathological studies have demonstrated reduced sweat gland nerve fiber density (SGNFD) and abnormal morphology in the upper extremities of ALS patients, contributing to sweating dysfunction [20, 22, 28–30]. SGNFD is also correlated with scores on the Small Fiber Neuropathy Symptom Inventory Questionnaire (SFN-SIQ) and the Visual Analogue Scale (VAS). ALS patients tend to have higher SFN-SIQ and VAS scores, which may reflect loss of SGNFs [22]. A related study reported a loss of pilomotor nerve fiber density (PNFD), along with morphological changes, such as axonal rupture and varicosities in the epidermis and basement membrane of ALS patients [31]. Sensory nerve fiber damage has also been reported in ALS patients who experience symptoms like numbness and pain [22]. Quantitative sensory testing (QST)—which can detect small nerve fiber damage and objectively assess sensory impairment—has shown that patients with spinal-onset ALS have elevated warm detection thresholds and reduced cold detection thresholds [23]. Additionally, skin biopsies have revealed reduced intraepidermal nerve fiber density and sensory axonal lesions in both ALS patients and mouse models [22, 32–34]. Isolated and chain-like swellings of nerve fiber axons in the epidermis have also been observed in early ALS stages [35]. Such axonal swelling, which results from disrupted axonal transport, may serve as an early marker of nerve fiber damage [36]. Together, these findings support the presence of nerve fiber injury and hypodensity in the skin of patients with ALS.

### Thickening walls of small blood vessels in the skin

Changes in the small blood vessels of the skin have been reported in ALS patients. Histopathological examination has revealed thickening of the dermal vessel walls in patients with sALS, while further electron microscopic analysis showed onion-skin-like morphological changes caused by β-amyloid deposits and basement membrane duplications within small vessels [37, 38]. The vascular bed was evaluated by measuring the vascular area relative to the dermal area. Confocal microscopy indicated that dermal vascular wall thickening in ALS patients may reduce the overall area of the vascular bed. The vascular network in the papillary dermis is denser and more complex in both hairless and hairy skin, a difference that may be related to autonomic innervation [31]. Additionally, some amorphous, fine particle-like substances deposited in the dermis may act as pressure absorbers, preventing blood vessels from becoming occluded [19]. These unique features of the small blood vessels may contribute to the skin’s ability to resist pressure ulcer formation in ALS. Mutations in the angiogenin (ANG) gene are among the factors implicated in the pathogenesis of ALS. ANG is a secreted ribonuclease involved in angiogenesis and the maintenance of vascular stability. In the skin, ANG is primarily produced by keratinocytes and endothelial cells. Immunohistochemistry studies have shown that ANG expression in the skin of sALS patients progressively decreases with disease progression, particularly in the nuclei of epidermal cells [39]. This reduction in ANG expression may impair blood vessel formation and branching. However, it remains unclear whether vascular changes occur in the skin of patients with ANG mutations, highlighting the need for further investigation.

## Abnormal deposition of genetically determined ALS-related proteins in skin tissue

**Figure 2.**
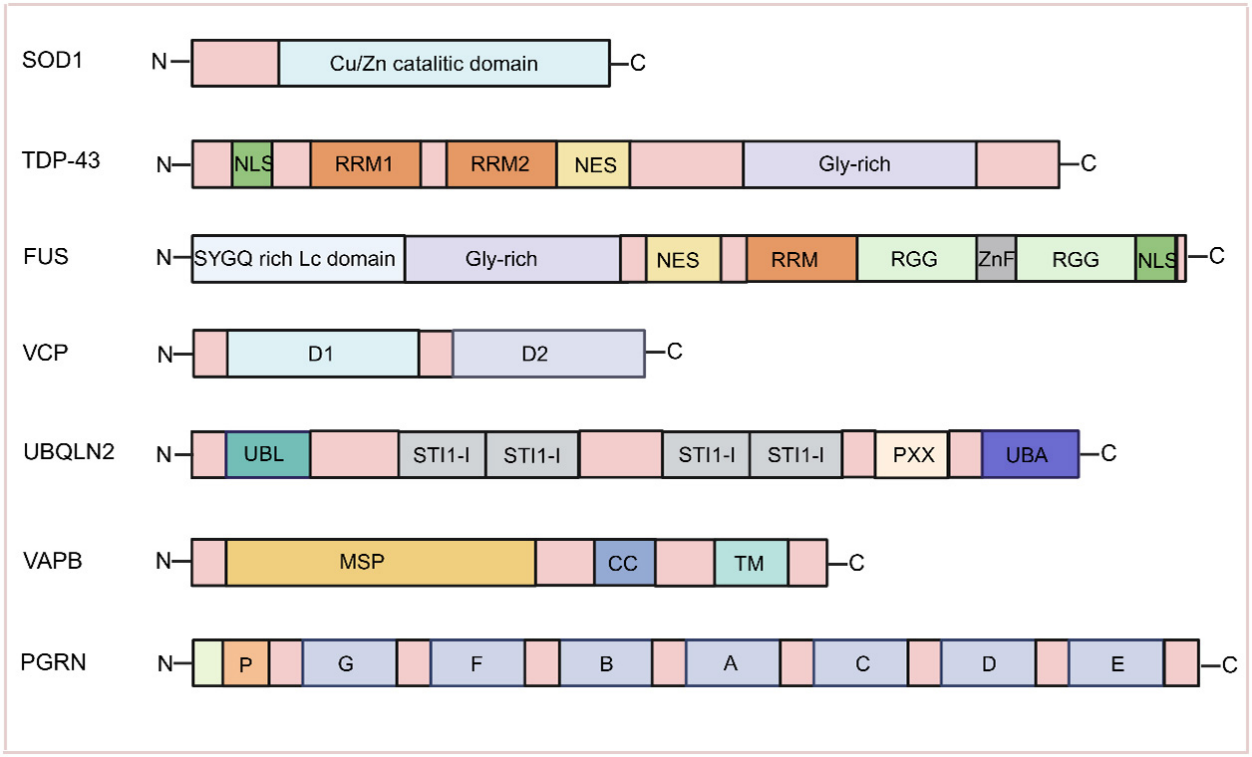
**Structures and domains of SOD1, TDP-43, FUS, VCP, UBQLN2, VAPB, and PGRN proteins (Created by BioRender.com).** Abbreviations: SOD1: Superoxide dismutase 1; FUS: Fused in sarcoma; VCP: Valosin-containing protein; UBQLN2: Ubiquilin 2; VAPB: Vesicle-associated membrane protein-associated protein B; PGRN: Progranulin; TDP-43: TAR DNA-binding protein 43; MSP: Major Sperm Protein; NLS: Nuclear localization signal; RRM: RNA recognition motif.

Aberrant aggregation of insoluble proteins, such as superoxide dismutase 1 (SOD1), TAR DNA-binding protein 43 (TDP-43), and fused in sarcoma (FUS) in the cytoplasm of MNs is a key pathological feature of ALS [40]. SOD1 is an antioxidant enzyme that protects cells from oxidative damage. It is a homodimer composed of 153 amino acids per monomer and contains binding sites for copper and zinc atoms (Figure 2) [41]. In ALS patients, mutant SOD1 proteins tend to aggregate in the cytoplasm, forming insoluble protein inclusions [42]. TDP-43 and FUS are two RNA-binding proteins (RBPs) primarily located in the nucleus. TDP-43 contains an N-terminal region, a nuclear localization signal (NLS), two RNA recognition motifs (RRM1 and RRM2), and a glycine-rich low-complexity domain (LCD) at the C-terminus. The FUS protein, composed of 526 amino acids, shares a similar domain structure with TDP-43 (Figure 2) [41]. In ALS, both proteins abnormally relocate from the nucleus to the cytoplasm and form cytoplasmic inclusion bodies, a process exacerbated by disease-associated mutations [40]. Moreover, TDP-43 and FUS are strongly associated with stress granules (SGs) [43]. SGs are dynamic, membrane-less cytoplasmic assemblies that form under cellular stress and consist mainly of translation factors, mRNAs, and RBPs. They regulate mRNA translation during stress and facilitate the resumption of protein synthesis once stress subsides, supporting the recovery of cellular functions [44]. In ALS, however, TDP-43 and FUS enter SGs and form insoluble aggregates, ultimately contributing to neuronal degeneration [43]. The protein quality control system plays a crucial role in degrading misfolded proteins and maintaining cellular protein homeostasis. The ubiquitin-proteasome system (UPS) is a central component of this process. UPS dysfunction can lead to protein aggregation [45]. Interestingly, at least three genes—valosin-containing protein (VCP), ubiquilin 2 (UBQLN2), and vesicle-associated membrane protein-associated protein B (VAPB)—have been implicated in protein quality control in the endoplasmic reticulum (ER) in the context of ALS. Accumulation of misfolded or improperly assembled proteins in the ER activates the ER-associated degradation (ERAD) pathway, resulting in the ubiquitination of these proteins for subsequent degradation via the UPS or autophagy pathways [46]. Mutations in VCP, UBQLN2, and VAPB impair ERAD function, promote protein aggregation, and contribute to MN degeneration. Additionally, abnormal deposition of certain genetically linked ALS-associated proteins has been observed in the skin of ALS patients (Table 1) [47, 48].

**Table 1:** Abnormal deposition of SOD1, TDP-43, FUS, VCP, UBQLN2, VAPB, and PGRN proteins present in the skin of ALS patients

**Proteins**	**Aggregation structure**	**Aggregate location**	**Sample type**	**References**
SOD1	Cytoplasmic aggregates	Skin fibroblasts	FALS cases (SOD1 mutation)	[49]
TDP-43	Cytoplasmic aggregates	Skin fibroblasts, skin tissue	SALS, fALS cases (TARDBP mutation)	[52–56]
FUS	Cytoplasmic aggregates	Skin fibroblasts	SALS, fALS cases (FUS mutation)	[47]
VCP	VCP+ cells	Skin tissue	SALS cases	[61]
UBQLN2	Ubiquitinated inclusions	Skin fibroblasts	FALS cases (UBQLN2 mutation)	[56, 64]
VAPB	Misfolded aggregates	Skin fibroblasts	SALS cases	[63]
PGRN	Diffuse or tightly aggregated granular PGRN+	Skin tissue	ALS cases	[69]

Abbreviations: SOD1: Superoxide dismutase 1; FUS: Fused in sarcoma; VCP: Valosin-containing protein; UBQLN2: Ubiquilin 2; VAPB: Vesicle-associated membrane protein-associated protein B; PGRN: Progranulin; TDP-43: TAR DNA-binding protein 43; fALS: Familial amyotrophic lateral sclerosis; sALS: Sporadic amyotrophic lateral sclerosis.

### SOD1

Only one study has investigated SOD1 protein expression in fibroblasts from fALS patients carrying the SOD1-V14M, SOD1-G16A, and SOD1-C111Y mutations using immunofluorescence staining. The results showed that SOD1 protein was diffusely aggregated in the cytoplasm, with reduced nuclear distribution. The cytoplasmic-to-nuclear ratio of SOD1 aggregates increased by 2.54-, 2.80-, and 3.25-fold for each respective mutation [49]. These findings suggest that skin fibroblasts from patients with SOD1 mutations may reflect key pathological features of ALS.

### TDP-43

Several studies have shown significantly elevated levels of TDP-43 in the skin of ALS patients [22, 50]. While TDP-43 mRNA levels in the skin decrease as the disease progresses, TDP-43 protein expression is increased in ALS patients with upper extremity onset compared to those with medullary or lower extremity onset. This suggests that the autoregulation of TDP-43 expression is disrupted in the skin of ALS patients, which may serve as a potential biomarker for early diagnosis of the disease [51]. Additionally, markedly elevated TDP-43 protein levels and abnormal aggregate formation have been observed in the skin of fALS patients with the TDP-43ˆA315T mutation, as well as in fibroblasts from ALS patients. These changes are accompanied by increased expression of ER stress-related proteins—GRP-78, ERK1/2—and the autophagy marker LC3, indicating dysregulation in both production and degradation of TDP-43 protein in the skin [52–56]. However, some studies have reported no detectable abnormal localization or aggregation of TDP-43 in the skin fibroblasts of sALS patients [57]. Given the small sample sizes in current studies, further multicenter research is necessary to determine whether changes in TDP-43 expression in the skin can reliably aid in ALS diagnosis.

### FUS

Immunohistochemical staining revealed that both the proportion and optical density of FUS-positive cells were significantly higher in the epidermis of sALS patients compared to controls [48]. This suggests that metabolic changes involving FUS may be present in the skin of ALS patients and could be associated with the disease process. Most mutations in the FUS gene are located at the C-terminus, which contains the NLS domain; these mutations may lead to the formation of cytoplasmic aggregates of mutant FUS proteins and their incorporation into SGs [58]. The subcellular localization of FUS in skin fibroblasts from controls, sALS patients, and asymptomatic ALS patients carrying the FUS P525L mutation was further investigated using immunocytochemistry and Western blotting. Results showed that nuclear FUS expression was present in fibroblasts from all groups, while cytoplasmic FUS expression was notably stronger only in ALS patients with the FUS P525L mutation. Upon exposure to two stressors—heat shock and dithiothreitol (DTT)—wild-type FUS from sALS patients and mutant FUS from P525L patients redistributed to the cytoplasm and were recruited into SGs. However, compared to controls and sALS patients, fibroblasts from FUS P525L patients exhibited a greater number of SGs that persisted longer, suggesting a pre-aggregated state. These molecular alterations may represent early pathogenic events that precede disease onset [47]. Nonetheless, some studies have reported no detectable changes in FUS expression patterns in skin fibroblasts from sALS patients [59]. Therefore, further research is needed before abnormal FUS deposition in the skin can be considered a reliable biomarker for ALS diagnosis.

### VCP

VCP is a member of the AAA family of adenosine triphosphatase (ATP)-associated enzymes, and its structure primarily comprises two AAA ATPase domains, D1 and D2, which together form a cyclic hexameric structure (Figure 2) [60]. Increased expression of VCP has been reported in the skin of patients with sALS. A large number of VCP-positive (VCP+) cells have been observed in the epidermis of ALS patients, with higher optical density compared to controls. Moreover, a significant positive correlation has been found between VCP immunoreactivity and disease duration in ALS patients [61]. These changes in VCP protein expression in the skin may be associated with the underlying disease process and could serve as a potential biomarker for ALS disease monitoring.

### UBQLN2

Skin biopsies from sALS patients showed a higher number of ubiquitin-positive cells in the epidermis compared to controls, with this change becoming more pronounced as the disease progressed [62]. UBQLN2 belongs to the family of ubiquitin-like proteins and contains an N-terminal ubiquitin-like domain (UBL) and a C-terminal ubiquitin-associated domain (UBA), which bind to ubiquitinated proteins and facilitate their degradation by the proteasome. It also includes four heat shock chaperone-binding motifs (STI1) and PXX repeats (Figure 2) [63]. UBQLN2-positive aggregates have been observed in the MNs of ALS patients, indicating impairment of the UPS. One study reported an increased number of ubiquitin-positive aggregates in the cytoplasm of fibroblasts from ALS patients with a UBQLN2 gene mutation after treatment with MG-132, a proteasome inhibitor [64]. These findings suggest that the skin of ALS patients may exhibit signs of impaired proteasomal degradation, resulting in the accumulation of ubiquitinated proteins. Therefore, similar to VCP, ubiquitin may also serve as a potential marker for ALS disease monitoring.

### VAPB

VAPB belongs to a family of vesicle-associated membrane proteins characterized by an N-terminal Major Sperm Protein (MSP) domain, a coiled-coil (CC) region, and a C-terminal transmembrane (TM) helix (Figure 2) [65]. In patients with autosomal dominant ALS caused by a point mutation in VAPB, aggregation of the mutant protein leads to structural reorganization of the ER. Similar alterations have been observed in peripheral blood mononuclear cells (PBMCs) from patients with sALS, suggesting their potential use as biomarkers for ALS research. In line with this, flow cytometric analysis of fibroblasts from sALS patients using an anti-human VAPB monoclonal antibody revealed reduced VAPB fluorescence. Immunofluorescence analysis with a polyclonal anti-human VAPB antibody showed spherical VAPB aggregates. Both findings likely reflect VAPB misfolding, and the observation that these aggregates co-localize with the ER chaperone GRP78 supports the idea that VAPB aggregation may cause ER damage [66]. Therefore, VAPB alterations in skin cells may serve as a potential biomarker for ALS diagnosis.

### Progranulin (PGRN)

PGRN is a secreted growth factor encoded by the GRN gene, which consists of approximately 593 amino acids and includes seven semi-repeated cysteine-rich motifs (P-G-F-B-A-C-D-E) (Figure 2) [67]. Nonsense or deletion mutations in this gene have been associated with ubiquitin-positive, tau-negative frontotemporal dementia (FTD-U) [68]. A study examining PGRN levels in the skin of patients with sALS revealed large amounts of poorly defined diffuse or tightly aggregated granular PGRN+ material in the epidermal and dermal glands. Furthermore, a significant positive correlation was found between the proportion of PGRN+ cells in the epidermis and disease duration in ALS patients [69]. These findings suggest that PGRN-related pathological changes may be detectable in the skin of ALS patients and could correlate with disease progression. However, additional studies with larger sample sizes are needed to validate these observations.

## Abnormal mitochondrial morphology and altered oxidative phosphorylation in skin cells

Mitochondrial dysfunction plays a key role in the pathogenesis of ALS. It contributes to severe oxidative stress, disruption of cytosolic calcium homeostasis, and activation of inflammatory responses due to the abnormal release of mitochondrial DNA—factors that may underlie MN death in ALS patients. Evidence of mitochondrial damage within MNs has been observed at early stages of the disease [70]. In patients with sALS, the mitochondria within keratin-forming cells near the basal layer of the skin show reduced long-axis length, area, and circumference. Electron microscopy further reveals outer membrane rupture and cristae degradation [71]. Similarly, in fALS, skin-derived fibroblasts exhibit disrupted mitochondrial dynamics, including imbalances in fission and fusion, reduced mitochondrial numbers, and compromised cristae integrity [72]. The primary function of mitochondria—ATP production through oxidative phosphorylation—is impaired in both sALS and fALS. Fibroblasts from these patients display reduced mitochondrial membrane potential, impaired oxidative phosphorylation, decreased ATP levels, increased reactive oxygen species (ROS) production, and redox imbalance [49, 70, 73]. However, one study involving only six sALS samples reported no structural changes in the mitochondrial network or significant alterations in ROS production [57]. Such discrepancies may reflect the heterogeneity of ALS in terms of disease duration, onset site, and other variables. As a result, whether mitochondrial alterations in the skin cells of ALS patients can serve as reliable biomarkers for disease monitoring remains an open question and warrants further investigation.

## Dysregulation of the inflammatory response in the skin

Inflammation plays a key role in the pathogenesis of ALS, contributing to neuronal damage and disease progression. Early autopsy studies revealed the presence of activated microglia, astrocyte proliferation, and lymphocyte infiltration in brain and spinal cord tissue from ALS patients [74]. More recently, some research teams have used PET imaging to detect microglial activation in regions, such as the motor cortex, anterior frontal lobe, thalamus, and pons during the early stages of ALS development [75]. Additionally, systemic inflammatory markers and immune cell populations in the blood of ALS patients show consistent alterations compared to healthy individuals. Notably, changes in the levels of neutrophils, CD4 and CD8 lymphocytes, and CD16 monocytes have been observed, correlating with disease severity [76, 77]. Dysregulation of neuroinflammatory responses—such as an increase in activated microglia and a reduction in regulatory T lymphocytes (Tregs)—has also been observed in ALS animal models like the SOD1 G93A mouse. Infusion of Tregs has been shown to suppress inflammation, prolong survival in these models, and lower peripheral levels of inflammatory markers such as acute-phase proteins (APPs) in patients [78, 79]. Some studies have found evidence of immune dysregulation in the skin of ALS patients. The tuberculin skin test following Bacillus Calmette–Guérin (BCG) vaccination is a standardized measure of adaptive immune function involving key ALS-associated immune cells like Tregs, Th17 cells, and monocytes. A recent study reported that a weaker tuberculin response to BCG vaccination was associated with a long-term reduction in ALS risk, supporting the idea of an altered immunomodulatory response in ALS pathogenesis [80]. On Guam, ALS patients exhibited reduced Treg levels and diminished antigenic responses in skin tissue early in disease progression. The diameter of positive skin reactions (sclerotomies) was significantly smaller in ALS patients injected with Candida-purified protein derivatives and streptokinase–streptozotocin antigens into the forearms, compared to healthy controls [81]. Moreover, skin epidermal cells and macrophages can release inflammatory factors, such as interleukin-6 (IL-6) and tumor necrosis factor-alpha (TNF-α), contributing to the systemic immune-inflammatory response. Immunohistochemical studies have shown that IL-6 and TNF-α expression in the epidermis and dermis of ALS patients increases with disease progression, with diffuse, unstructured TNF-α+ particles accumulating in the epidermis. In response to inflammation and immune dysfunction, epidermal keratinocytes release TNF-α, which stimulates the expression of endothelial cell adhesion molecules, leading to the accumulation of inflammatory cells. The elevation of these inflammatory mediators may strengthen the skin’s defense mechanisms and could be linked to a lower incidence of decubitus ulcers in ALS patients [82, 83]. These findings suggest that abnormal immune responses in the skin may serve as systemic biomarkers associated with ALS progression.

## Other molecules change in ALS skin

In addition to the changes described above, several other molecular alterations have been identified in the skin of individuals with ALS. Elevated levels of specific substances were found in the epidermis, dermis, and glands of ALS patients, including matrix metalloproteinase 9 (MMP-9), insulin-like growth factor I (IGF-I), vascular endothelial growth factor (VEGF), hepatocyte growth factor (HGF), cystatin C, laminin 1, and HA [84–90]. Among these, MMP-9 belongs to the matrix metalloproteinases (MMPs) family, which is responsible for degrading extracellular matrix components such as collagen. Increased levels of MMP-9 have been observed in both the skin and spinal cord of SOD1ˆG93A mice, suggesting that MMP upregulation may serve as a link between neurons and the skin. This connection could help elucidate ALS pathogenesis and highlights MMP-9 as a potential biomarker for the disease [91]. IGF-I, laminin 1, and HA are thought to be involved in collagen degradation and synthesis. Additionally, IGF-I, VEGF, HGF, cystatin C, laminin 1, and HA have all been associated with ALS disease progression. Their increased immunoreactivity in the skin may also contribute to the observation that ALS patients are less prone to developing pressure ulcers. A reduction in galectin-1 immunoreactivity has also been observed in the dermis. Galectin-1 is a muscle cell-derived growth regulator that influences dermal fibroblast proliferation. It also plays a role in the formation of axonal spheroids, an early pathological feature of ALS. Notably, intramuscular injection of oxidized galectin-1 into ALS model mice has been shown to improve clinical outcomes and extend survival [92]. These molecules may serve not only as indicators of disease progression but also as potential therapeutic targets in ALS.

## Conclusion

Currently, identifying reliable biomarkers remains one of the major challenges in diagnosing ALS. Skin examinations in ALS patients are easy to perform and offer valuable insights into the disease’s pathogenesis. Tests assessing skin autonomic and sensory nerve function, skin biopsies analyzing structural changes, MMP-9 expression, and immune biomarkers such as tuberculin reactivity could serve as non-invasive tools for ALS detection and monitoring its progression. Therefore, large-scale prospective studies focusing on skin-related changes are still needed to identify and investigate pathological alterations in both skin structure and key molecular markers associated with ALS.

**Conflict of interests:** Authors declare no conflicts of interest.

**Funding:** This work was supported by the National Natural Science Foundation of China (82160320), the Central Government Guides Local Science and Technology Development Fund Projects (2021ZY0010), and the Natural Science Foundation of Inner Mongolia Autonomous Region of China (2022MS08048).
